# Supplementary material for: MetaRibo-Seq measures translation in microbiomes
Source: Nat Commun. 2020 Jun 29;11:3268. doi: 10.1038/s41467-020-17081-z (PMC7324362; doi:10.1038/s41467-020-17081-z)
Supplement: Supplementary file 10 — Supplementary Data 7 [file 41467_2020_17081_MOESM10_ESM.zip › File2/Confidence_High_Taxonomy/83710_out.krona.html]

Javascript must be enabled to view this page.

members
magnitude
magnitudeUnassigned
count
unassigned
taxon
rank

83710\_out

3

superkingdom
2
3

phylum
1239
3

class
186801
3

order
186802
3

3
family
186803

3
572511
genus

33038
species
3

SRS012273\_contig\_number\_47265SRS014948\_contig\_number\_contig-100\_566.107080SRS1055043\_contig\_number\_24149
